# Classification of Visual and Non-visual Learners Using Electroencephalographic Alpha and Gamma Activities

**DOI:** 10.3389/fnbeh.2019.00086

**Published:** 2019-05-07

**Authors:** Soyiba Jawed, Hafeez Ullah Amin, Aamir Saeed Malik, Ibrahima Faye

**Affiliations:** ^1^Centre of Intelligent Signal and Imaging Research, Universiti Teknologi PETRONAS, Seri Iskandar, Malaysia; ^2^Department of Electrical and Electronic Engineering, Universiti Teknologi PETRONAS, Seri Iskandar, Malaysia; ^3^Asia Pacific Neuro-Biofeedback Association, Singapore, Singapore; ^4^Department of Fundamental and Applied Sciences, Universiti Teknologi PETRONAS, Seri Iskandar, Malaysia

**Keywords:** EEG study, learning styles, visual learner, feature extraction, classification

## Abstract

This study analyzes the learning styles of subjects based on their electroencephalo-graphy (EEG) signals. The goal is to identify how the EEG features of a visual learner differ from those of a non-visual learner. The idea is to measure the students’ EEGs during the resting states (eyes open and eyes closed conditions) and when performing learning tasks. For this purpose, 34 healthy subjects are recruited. The subjects have no background knowledge of the animated learning content. The subjects are shown the animated learning content in a video format. The experiment consists of two sessions and each session comprises two parts: (1) Learning task: the subjects are shown the animated learning content for an 8–10 min duration. (2) Memory retrieval task The EEG signals are measured during the leaning task and memory retrieval task in two sessions. The retention time for the first session was 30 min, and 2 months for the second session. The analysis is performed for the EEG measured during the memory retrieval tasks. The study characterizes and differentiates the visual learners from the non-visual learners considering the extracted EEG features, such as the power spectral density (PSD), power spectral entropy (PSE), and discrete wavelet transform (DWT). The PSD and DWT features are analyzed. The EEG PSD and DWT features are computed for the recorded EEG in the alpha and gamma frequency bands over 128 scalp sites. The alpha and gamma frequency band for frontal, occipital, and parietal regions are analyzed as these regions are activated during learning. The extracted PSD and DWT features are then reduced to 8 and 15 optimum features using principal component analysis (PCA). The optimum features are then used as an input to the *k*-nearest neighbor (*k*-NN) classifier using the Mahalanobis distance metric, with 10-fold cross validation and support vector machine (SVM) classifier using linear kernel, with 10-fold cross validation. The classification results showed 97% and 94% accuracies rate for the first session and 96% and 93% accuracies for the second session in the alpha and gamma bands for the visual learners and non-visual learners, respectively, for *k*-NN classifier for PSD features and 68% and 100% accuracies rate for first session and 100% accuracies rate for second session for DWT features using *k*-NN classifier for the second session in the alpha and gamma band. For PSD features 97% and 96% accuracies rate for the first session, 100% and 95% accuracies rate for second session using SVM classifier and 79% and 82% accuracy for first session and 56% and 74% accuracy for second session for DWT features using SVM classifier. The results showed that the PSDs in the alpha and gamma bands represent distinct and stable EEG signatures for visual learners and non-visual learners during the retrieval of the learned contents.

## Introduction

Human being growing in modern societies are exposed to certain learning environment. The way an individual processes information contributes toward an individual’s learning ability (Kim et al., 2006). Many attributes are important in the learning scene, such as intelligence, learning environment, learning abilities (Stern, 2017) and learning style, among which learning style is the most consistently studied (Koć-Januchta et al., 2017; Yazici, 2017). Learning style is not a new concept, as it has been a topic of discussion for years (Koć-Januchta et al., 2017). Learning style is defined as an individual’s preferred way of learning (Plass et al., 1998).

Researchers associate learning styles with the patterns of information processing in the brain, known as cognitive styles. The benchmarks to differentiate between the learning style and cognitive style are defined as follows (Mayer and Massa, 2003): The style preferred by individuals for representing and processing information is defined as a learning style. However, the methods of representing and processing information by the brain are classified under cognitive style. Researchers hypothesize that a relation exists between the learning style and cognitive style. The outcomes of the existing research clearly shows that the information processing is linked to the preferred learning style of an individual (Ahn et al., 2010). Thus, the existing studies conclude that every individual has their own preferred learning style.

Learning style is further broken down into learning style models and learning style modalities (Ahmad and Tasir, 2013). A theoretical coherence and a common framework for all learning style models are lacking (Klašnja-Milićević et al., 2016). However, different studies reported that learning styles and preferences are constitutionally based on four learning modalities: visual (seeing), auditory (hearing), kinesthetic (moving), and tactile (touch) (Klašnja-Milićević et al., 2016). Learning using technology, for example videos, can make the learning process interesting and create an enjoyable experience for the students (Abid et al., 2016). These are the reasons why most educational models are based on visual and auditory modalities.

Further, according to statistics, 65% of the population is visual learners^1^ (Zopf et al., 2004). Visual learners learn by visual reinforcements, such as videos contents (see text footnote 1) (Zopf et al., 2004). Many researchers have explored only the visual verbal learning style of the visual modality; Felder’s theory claims that the major leaning style is visual. Felder’s theory has four dimensions. Each learner is characterized based on its preference for each of these dimensions. The first dimension discriminates between an active and a reflective way of processing information. The second dimension is sensing versus intuitive learning. The third, visual-verbal dimension differentiates learners who remember best and therefore prefer to learn from what they have seen (e.g., pictures, diagrams and flow-charts), and learners who get more out of textual representations, regardless of whether they are written or spoken. In the fourth dimension, the learners are characterized according to their understanding. Sequential learners learn in small incremental steps and therefore have a linear learning progress. In contrast, global learners use a holistic thinking process and learn in large leaps (Felder and Silverman, 1988).

Learning styles, such as visual learning style, are highly associated with brain patterns. Therefore, a person with a visual learning style is observed to have lower cognitive load when processing visual information. Visual learners are further categorized into visual/verbal learners: when an individual’s brain shows lower cognitive load while learning through written material, such as words, he/she is categorized as a verbal learner. Some interesting works have been carried out to investigate the visual verbal learning style of learners with hearing impairment. In such studies, researchers have primarily explored the difference between the visual and verbal learners, which are the two components of the visual learning modality, according to Felder’s theory (Marschark et al., 2013). Another study is conducted to investigate the visual attention of the learners when a lecture is delivered using a power point presentation. This study also investigates the visual and verbal aspects of the visual modality (Yang et al., 2013). In addition, another study (Kim et al., 2006) have reported that the number of visual learners is as high as 80% when compared to the verbal learners among college students. This study selected students with a visual learning style using the Felder–Silverman’s index of learning style (ILS) subjective measures. The above-mentioned studies are some of the examples of studies relating to visual learning and other brain activities using only subjective measures such as learning style test. The learning style tests involve self-estimation of learning style, which has bias in it. However, there exists a relationship between learning and working memory (Hindal et al., 2009). To do an independent analysis we explore this relationship to identify the learning style such as visual learners and non-visual learners of the participant. Here, in our experiment the control variable is Raven’s Advanced Progressive Matrices (RAMP) fluid intelligence test. This analysis is independent of learning tests purely looking at the brain patterns.

Suggesting learning style without considering brain pattern can increase the cognitive load. The cognitive load of the learner increased when information processing become complex for the learner. Knowing the learning style can optimized the learning and make it easier for students to understand the content. Also, it is possible one thinks that they have certain learning style such as visual, but in reality it’s just a learned behavior because that’s the only method of learning known to them which might not be according to their brain patterns, thus it’s important to find suitable learning style based on brain patterns.

The electroencephalogram (EEG) (Teplan, 2002) is one of the many tools that can be used for recording brain patterns while performing mental activities or while resting.

The focus of this study is to differentiate the visual learners from the non-visual learners using EEG. Here, we will discuss how the EEG recording is interpreted by the researchers. In general, the EEG signal is divided into five bands: alpha, beta, delta, theta, and gamma (Amin et al., 2017). In this study, the analysis is performed using the alpha and gamma bands. The analysis of theta and delta band is the part of our future work. The focus is on alpha and gamma because there exist a strong association between alpha waves and gamma waves and learning (Gruber et al., 2002; Grabner et al., 2017).

Alpha waves have a frequency range of 8–12 Hz. Changes in the alpha frequency are observed during visual learning tasks as well as during intelligence tasks. Alpha activity increases in the frontal region and decreases in the right parietal and right temporal regions during visual learning (Frederick et al., 2016; Tóth et al., 2017).

Gamma waves have a frequency range of 30–80 Hz (Tseng et al., 2016). Gamma waves are highly associated with high-level visual information processing, such as visual learning (Jia and Kohn, 2011). A decrease in the gamma band is observed in the frontal location during visual learning. Therefore, many existing studies are focused on gamma waves (Yao et al., 2017).

The objective of this study is to classify visual learner and non-visual learner. For classification some meaningful information is needed to be extracted from EEG recorded signals. Thus, features such as PSD, PSE, and DWT are extracted to feed into the classifier. We use: (1) PSD: which is useful when some key features need to be extracted from EEG data (Hamzah et al., 2016). The PSD feature is used by researchers (Amin et al., 2014) for cognitive task analysis such as visual learning. This feature is obtained for EEG data using the Welch technique and the Hamming window with 50% overlapping epochs (Amin et al., 2014). The analysis shows that a person with low intelligence has a higher value of the lower power of alpha and vice versa (Harmony et al., 1996; Jaušovec, 2000; Doppelmayr et al., 2002; Grabner et al., 2004; Thatcher et al., 2005; Micheloyannis et al., 2006; Huang and Charyton, 2008; Riečanský and Katina, 2010). (2) DWT features are suitable for non-stationary signals (Jahankhani et al., 2006). These features are robust enough and give discriminative information to distinguish the visual learners from non-visual learners. (3) PSE features has good effect for the change of non-linear dynamic states, it is suitable for small dataset which makes it suitable for EEG signals (Zhang et al., 2008). The autoregressive, adaptive autoregressive (Akhtar et al., 2012; Ali et al., 2016) are some of the other feature extraction methods for the non-stationary EEG signals.

The next step toward analyzing the brain waves is feature selection. There exist many feature selection techniques (Chandrashekar and Sahin, 2014), but due to the high dimensional nature of EEG datasets, dimension reduction technique such as PCA is used in this study for feature selection.

The selected features are then given to the classifier to classify visual learner and non-visual learner. In classification, a machine-learning algorithm is trained and tested using a certain amount of experimental data to develop a model for the new related data (Amin et al., 2017). The idea is to train the set of data having observations with a known category membership, using the features as independent variables to set the target between different feature spaces. To classify the EEG dataset, the classifier must be able to handle the following two issues: (1) Curse of dimensionality: Based on the amount of data that represents different classes, increases exponentially with the feature vector dimensionality. This occurs when the training data is small, and the feature vector is large; in this type of scenario, the classifier did not give good results. Therefore, it is recommended that training samples of the class is at least five times the training samples of the class of dimensionality (Lotte et al., 2007). In the case of the EEG dataset, the dataset is small compared to the high dimensionality of the feature vector, which leads to poor classification (Lotte et al., 2007). (2) The bias-variance trade-off: Bias is defined as the divergence between the estimated mapping and the possibly attained mapping. The bias is dependent on the training set. To classify the data with minimum error, the bias must be low. The stable classifier has a low variance and a high bias. The simple classifier has a low bias and a high variance, which renders the simple classifier more suitable for EEG datasets, as it outperforms the complex classifiers (Lotte et al., 2007; Córdova et al., 2015).

In this study, we present a method for classifying the visual learners from non-visual learners based on the EEG signals and employed the PSD and DWT as a feature extraction, the PCA as a feature selection technique, and the *k*-NN and SVM as a machine-learning algorithm for the classification of two groups, i.e., visual learners and non-visual learners. We attempt to explore the brain neuronal behavior of the visual learners as compared to non-visual learners when the information is presented according to their preferred learning modality.

The paper is organized as follows: Materials and Methods, Results, Discussion, Limitations of the Study, and Conclusion.

## Materials and Methods

This section comprehensively explains the overall process implemented for this study. From the EEG data, the pre-processing, feature extraction, feature selection and development of the brain model classifying the visual learners and non-visual learners using the *k*-NN and SVM classifier. The input to our system is an EEG signal. The next step is feature extraction, followed by feature selection and classification. Each block of the system starting from the data set is explained in detail below.

### Subjects

Thirty-four healthy university subjects (age: 18–30 years, 23.17 ± 3.04) were recruited for the experiment. All subjects had normal or “corrected to normal” vision. All subjects were free from neurological disorders and medications and did not have hearing impairments. All subjects are male. All subjects signed an informed consent document prior to the beginning of the trials. This study was approved by the Ethics Coordination Committee of the Universiti Teknologi PETRONAS (Amin et al., 2015b). The experimental procedure is same for all the participants.

### Raven’s Advanced Progressive Matrix (RAPM) Test

Raven’s advanced progressive matrix (RAPM) (Raven, 2000) is used to measure intellectual ability. It is a non-verbal test that commonly and directly measures two components of a fluid’s cognitive ability (Raven, 2000) defined as: (i) “the ability to draw meaning out of confusion,” and (ii) “the ability to recall and reproduce information that has been made explicit and communicated from one to another.” It has 48 series of patterns that are further divided into two sets (I and II): One for practice and the other is to assess cognitive ability. Set I have 12 patterns that are used for practice. However, Set II has 36 patterns that are used to measure cognitive ability.

**Figure 1:**
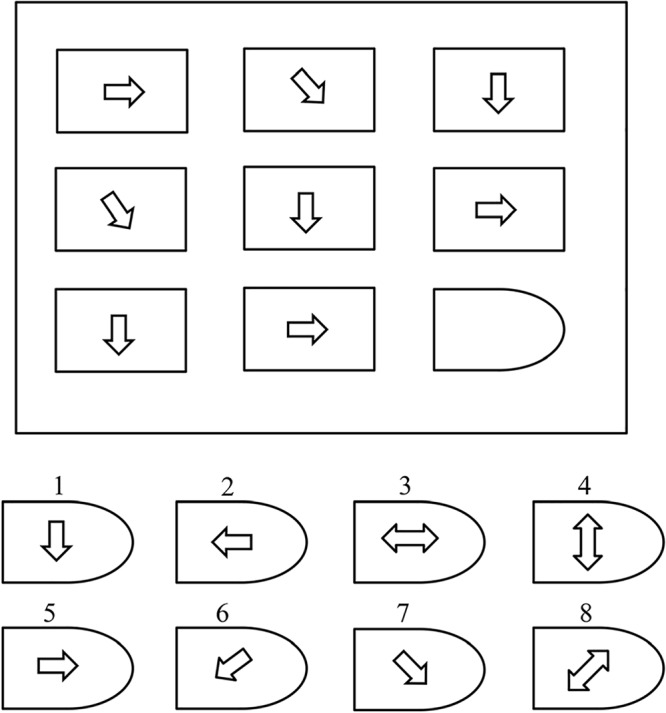
Example of RAPM problem (Amin et al., 2015a).

The pattern of the test consists of a 3 × 3 cell structure representing a certain geometrical shape, except the bottom-right cell, which is empty, as shown in Figure 1. Eight options are available for the empty cell. For each correct answer the user gives a score “1” and a “0” for each incorrect answer. Users are given 10 min to complete Set I and 40 min to complete Set II (Raven, 2000). We use RAMP memory test as we know, Memory plays an important part in learning as in the learned information is stored in memory. Memory is the expression of what one has learned. Thus, learning and memory are related (Passolunghi and Costa, 2019).

### Tasks

**Figure 2:**
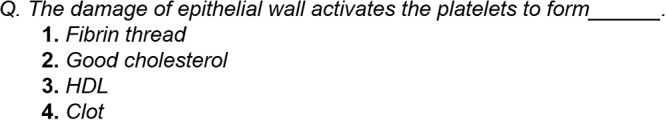
Example of multiple-choice question (Amin et al., 2015b).

Two main tasks are involved: (1) the learning task, and (2) memory or information retrieval task. The material use for learning task was based on biological contents related to human anatomy. The biological content was taken from commercially available high standard secondary curriculum (grade 11∼12). The content has high quality computer animations related to the complex human anatomy concepts, functions and diseases. The duration of this animated learning material is 8–10 min. The subjects had backgrounds in mathematics and engineering and had no prior knowledge of the learning content. Therefore, this selected learning content provide new information to the subjects and is suitable for the assessment of memory skills and learning. In addition to learning task a memory retrieval task was prepared. In the memory retrieval task, 20 multiple-choice questions (MCQs) that include the learned animated contents, were presented (see Figure 2). Each MCQ has four possible answers, out of which one is correct. The time to answer each MCQ is 30 s within a maximum time limit of 10 min. Subjects were asked to press a numeric key on the keyboard, serially numbered #1 to #4 corresponding to each possible answer.

### Procedure

The subjects did the test in the following order. First RAMP was done. The subjects were divided into two groups based on their RAMP score. Which is explained in detail in experimental results section. The next step was eyes open/eyes close test. The third step was showing subjects the learning content. After 30-min break retrieval task was done. The second retrieval task was done after 2 months. The EEG was recorded during eyes open, eyes close, learning task and retrieval task. The retrieval task session done 30 min after learning task session is named as recall session 1 and recall session 2 is the retrieval task done 2 months after recall session 1.

To ensure that subjects did not have any background knowledge a pre-test was conducted where the subjects were asked to solve 10 questions related to learning the animated content. The exclusion criteria were based on the results of the pre-test; if the subject manages to answer more than 10% of the pre-test questions correctly, he was excluded from the experiment. Each subject was briefed on the procedure. At the end of the learning session, a 30-min break was given, after which the subjects took retrieval task to assess their learning performance. Each learning task was presented on a 42-inch TV screen at 1.5 m from the subject. All tasks were implemented with the E-Prime Professional 2.0 (Psychology Software Tools, Inc., Sharpsburg, PA, United States) (Schneider et al., 2002).

### Electrophysiological (EEG) Recording

**Figure 3:**
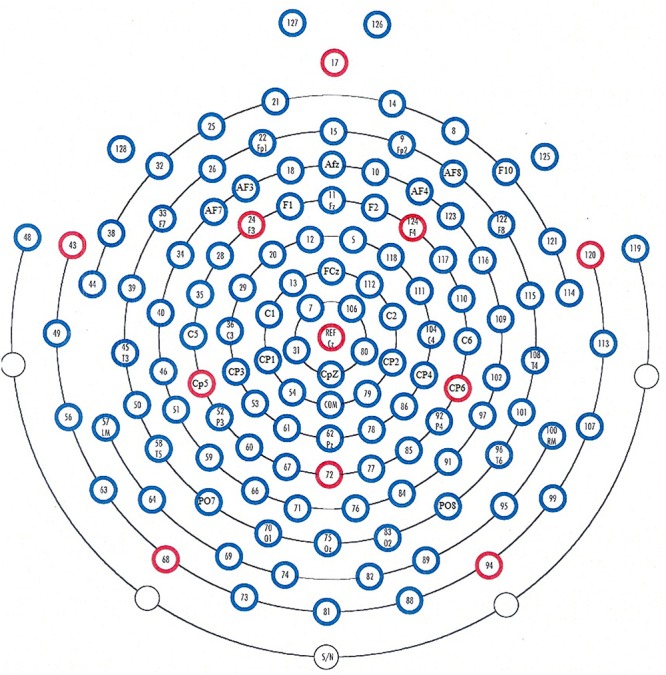
Placement of electrodes (HydroCel Geodesic Net 128 channels with Cz as a reference).

The EEG continuously recorded the subject’s responses via 128 scalp loci using the HydroCel Geodesic Sensor Net (Electrical Geodesic Inc., Eugene, OR, United States) (shown in Figure 3). The notch filter is applied during EEG recording, to eliminate the power-line noise in the recorded EEG. All electrodes referenced a single vertex electrode, Cz (which is the standard configuration of the net), from which raw signals were amplified with the EGI NetAmps 300 amplifier’s band-pass filter (0.1–100 Hz). The impedance was maintained below 50 kΩ, and the sampling rate was 250 Hz. Impedances indicate electrode performance, it’s better when lower impedance value and poorer when higher impedance value. The high values of impedance introduce noise in the EEG signals that is why it is recommended to use low impedance values. To record good quality noise free EEG, the electrodes of EEG cap must have impedances within certain range. The range is defined as follow (0–50 kΩ) good quality, (50–100 kΩ) acceptable range, (100–200 kΩ) high value. We record EEG within the range of (0–50 kΩ) by keeping the impedances below 50 kΩ.

### Behavioral Data Analysis

The behavioral data (performance of the subjects) is analyzed to determine the accuracy of the information retrieved by the subjects after the learning and retrieval tasks using the visual contents. The subjects are asked 20 questions of 1-min duration. The total length of time window is 20 min × 60 s = 1,200 s. The assessment of the learning performance are based on the correct responses and the reaction time per question for each subject. The reaction time reflects the information processing speed based on intelligence. The learning performance is then measured based on the percentage of correct responses.

### Pre-processing

After the recording of raw EEG data, each subject’s continuous EEG data was pre-processed with NetStation v4.5.4 (Electrical Geodesic, Inc., Eugene, OR, United States). A brief description of the pre-processing is provided here. (a) A band pass IIR filter was applied (0.5–48 Hz, roll off 12 dB octave) to remove DC components and high frequency muscular artifacts. (c) The artifacts such as eye movement and muscle movement are corrected using the surrogate model approach of the BESA software (Roberts et al., 2010). Bad channels were discarded from the segments. The clean EEG is then exported to MATLAB for further processing.

### Feature Extraction Methods

The relevant information extraction from raw signals is a critical step in the EEG pattern classification, owing to its direct influence on the classification performance. In this study, the PSE, PSD and DWT methods were used to extract the EEG features from frontal, occipital and parietal region for the classification of visual learners from non-visual learners. The clean EEG signals are then divided into the alpha (8–13 Hz), beta (13– 28 Hz), theta (4–8 Hz), and delta (0.5–4 Hz) and gamma band (25–100 Hz) frequency bands.

The PSD is computed using the FFT with the Welch method and hamming window to estimate the power spectrum of the EEG time series (Welch, 1967) with 2-s segments (2 × 250 = 500 points), 50% overlapping (250 points) and kept the nfft as 512 points. In addition, the PSE is obtained by implementing the procedure mentioned in Zhang et al. (2008).

Discrete wavelet transform is another feature that is extracted. DWT is famous method for EEG non-stationary signals. It’s an estimation technique where wavelet function is used to represent the signal as an infinite series of wavelets. Based on mother wavelet, the signal is a linear combination of wavelet functions and weighted wavelet coefficients (Akhtar et al., 2012).

The features are extracted from frontal, occipital and parietal regions of brain. ANOVA is applied using Matlab to see statistically the variance of features. The *P*-value for power spectral density of alpha and gamma is *p* < 0.05 showing statistically independence of two groups. That is why we choose PSD of alpha and PSD of gamma, DWT of alpha and DWT of gamma for further analysis. The total number of computed PSD features for two sessions are 34 × 16 = 544 and 31 × 16 = 496. And the total number of computed features for DWT for two session are 34 × 29 = 986 and 34 × 29 = 899.

### Feature Selection

The features extracted above show the discriminative information that is used for further analysis. The feature design for this EEG study is not a straightforward task. It has challenges such as a noisy environment, multiple sources, and overlapping due to multi-tasking in the brain. The EEG signals have poor signal-to-noise ratios. The curse of dimensionality is also present. Because of the above-mentioned challenges, feature selection is important. There are many methods for feature selection (Chandrashekar and Sahin, 2014). For this study the features are selected using the PCA (Dong and Qin, 2018) to deal with the challenge of curse of dimensionality. The PCA can be obtained by using the following steps:

(1) The first step is data normalization performed by subtracting the mean values from the columns.
(2) The covariance of the normalized data is then calculated.
(3) The eigenvector and eigenvalues can be calculated from the covariance matrix.
(4) A vector is obtained that consists of eigenvectors.
(5) The principal component is obtained by multiplying the transpose of the selected feature vector with the original data.
(6) The selected feature vector is obtained by taking the maximum of the principal component which corresponds to largest eigen values in the data.

The extracted features are the PSD of size [34 × 16] and DWT of [34 × 29]each. However, using the PCA, the selected feature vector is of size [34 × 8] and [34 × 15]. PCA is used with 99% variance. These 8 and 15 features are representing 99% variance so there is no need to add other features.

### Brain Learning Model Using Classifiers

To distinguish the visual learners from the non-visual learners, a brain-learning model is developed using the *k*-NN and the SVM. The *k*-NN is a widely used technique for classification problems. In the *k*-NN, the *k* value is the value of the nearest neighbor. *k* is non-parametric; the rule of the thumb of choosing the *k* value is $k\;=\;\sqrt{\frac{N}{2}}$ where *N* is the number of samples (Altman, 1992). The idea is to distinguish the visual learners from the non-visual learners and therefore, we set the value of *k* = 3. The *k* value plays an important role as it draws a boundary that segregates the visual learners from the non-visual learners. For the *k*-NN, several options are available for the distance metric. However, for this model, the Mahalanobis distance metric is used. Since the EEG dataset is non-linear, the distance metric such as the Euclidian distance does not give good results as the Euclidean distance metric is more suitable for linear datasets. However, the Mahalanobis distance formula is the same as the Euclidian distance having a covariance parameter, which makes it a more suitable and practical option for data with non-linearities. The formula for the Mahalanobis distance is presented in equation.

$$D\;=\;\sqrt{{\left(x\;-\;\mu \right)}^{\text{T}}S^{-1}(x\;-\;\mu )}$$

Here, *x* is a set of observations, where μ is the mean of the observations and *S* is the covariance matrix.

The SVM classifier is best suitable for binary classifications. It classifies data by finding the best hyperplane that separates all data points of one class from other glasses. The best suitable hyperplane is the one with the largest margin (Witten et al., 2016). SVM is another classifier used for this study.

The step taken before the model development is the randomization of data into two parts, here 10-fold cross validation is used for training and testing. The training set was used to train the model; however, testing is performed to evaluate the overall ability of the dataset’s training part. The above-mentioned method is the standard practice in machine learning used by many researchers in their work (Zhang and Zhou, 2007).

## Results

The behavioral data is analyzed to measure the performance of the visual learners and non-visual learners. For learning, the correct responses and reaction times are computed for each participant. The reaction time shows the mental speed of information retrieval and is measured from the point where the MCQ is displayed until the participant presses a button for the selection of an answer. The percentage of correct responses per participant was then used to measure his learning performance. The total number of trials available per subject was 34 subjects × 20 MCQ = 680 trials. To assess the learning ability RAMP score is used. The subjects are divided into two equal groups using median score (Amin et al., 2015b). Based on the median score, the subjects who scored equal or above the median are considered as visual learners and those who scored less than the median are considered as non-visual learners. To classify the visual learner and non-visual learner, we analyze the retrieval task, the first retrieval task is recorded 30 min after the learning task (recall session 1) and the second retrieval task is recorded 2 months after the learning (recall session 2). To classify visual learners from non-visual learners the suitable features are selected, which can discriminate the two groups. To measure the statistical significance of the features statistical analysis is performed. First the features are extracted from frontal, occipital, and parietal regions of brain. The total number of computed features for two sessions (recall session 1 and recall session 2) are 34 × 16 = 544, 31 × 16 = 496 and 34 × 29 = 986, 31 × 29 = 899. Then one-way ANOVA with Tukey *post hoc* test is performed on extracted features. The results of one-way ANOVA show *P*-value for PSD of alpha and PSD of gamma, DWT of alpha and DWT of gamma (*p* < 0.05) which shows statistically independence of two groups. However, the *P*-value of PSE did not show statistically significance because of that reason we use PSD and DWT feature for further analysis. For feature selection, the PCA is used to best describe the variance in the features and to reduce their dimensionality. We prefer PCA over LDA, because PCA perform better in case where number of samples per class is less. Whereas LDA works better with large dataset having multiple classes^2^.

**Figure 4:**
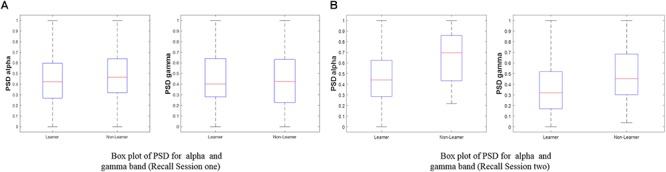
Box plot of PSD for (i) alpha (ii) gamma with respect to the visual learner and non-visual learner groups. The focus of our study is the left and right brain hemispheres during the learning state. **(A)** Recall session 1 (*N* = 34). **(B)** Recall session 2 (*N* = 31).

To evaluate the model performance, the accuracy, sensitivity (true positive rate), and specificity (true negative rate) parameters are computed, and the receiver operating characteristic (ROC) curve is obtained. To calculate the accuracy, sensitivity, and specificity, the confusion matrix is first computed. A confusion matrix is used to describe the performance of a classification model on a set of data with known true values. Figure 4 shows the box plot indicating the significant pattern between a visual learner and a non-visual learner for the alpha and gamma bands of recall session 1 and recall session 2. The distinct mean level can be observed for the two groups, where the mean of the non-visual learner is higher than the mean of the visual learner for the alpha sub-band, which is similarly observed for the gamma band case.

Tables 1A,B show the average of the confusion matrix for the PSD of alpha and gamma waves obtained from different iterations, respectively. From Tables 1A,B, two predicted classes for the visual learner (L) and non-visual learner (NL) with *n* = 34 can be seen, meaning that 34 subjects were tested. From the results, we observed that the classifier predicted the Ls 16 times and the NLs 1 time for the alpha waves. For the gamma waves, the predicted Ls are 16 and the NLs are 1. However, there are actually 17 Ls and 17 NLs, where TP is a true positive, TN is true negative, FP is false positive, and FN is false negative. The TPs are the instances where the predicted visual learners are actually visual learners. The TNs are the instances where the predicted non-visual learners are actually non-visual learners. The FPs are when the visual non-learners are predicted as visual learners. The FNs are when the visual learners are predicted as non-visual learners.

**Table 1:** Confusion matrix and performance matrix of PSD of alpha and gamma band using *k*-NN classifier (recall session 1).

						(C) Performance matrix: specificity, sensitivity, and			
						accuracy for alpha and gamma waves to distinguish			
(A) Confusion matrix of alpha			(B) Confusion matrix of gamma			visual learners from non-visual learners			
*n* = 34	Predicted (NL)	Predicted (L)	*n* = 34	Predicted (NL)	Predicted (L)		Specificity (%)	Sensitivity (%)	Accuracy (%)
Actual (NL)	TN = 16	FP = 1	Actual (NL)	TN = 16	FP = 1	Alpha waves	94%	94%	97%
Actual (L)	FN = 1	TP = 16	Actual (L)	FN = 1	TP = 16	Gamma waves	94%	94%	94%

From the confusion matrix shown in Tables 1A,B, the accuracy, sensitivity, and specificity are calculated. Mathematically, the accuracy, sensitivity, and specificity parameters are shown in equations.

$$Accuracy\;=\;\left(\frac{TP\;+\;TN}{TP\;+\;TN\;+\;FP\;+\;FN}\right)\;\times \;100\%$$

$$Sensitivity\;=\;\left(\frac{TP}{TP\;+\;FN}\right)\;\times \;100\%$$

$$Specificity\;=\;\left(\frac{TN}{TN\;+\;FP}\right)\;\times \;100\%$$

Table 1C shows the specificity, sensitivity, and accuracy corresponding to visual learners and non-visual learners for the PSD of alpha and gamma waves.

**Figure 5:**
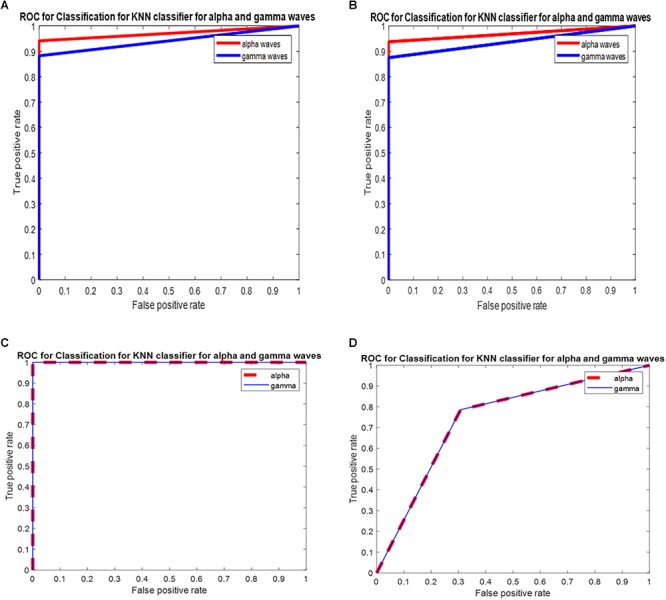
**(A)** ROC for classification for *k*-NN classifier for PSD of alpha waves and gamma waves with AUC of 0.97 and 0.94 (recall session 1). **(B)** ROC for classification for *k*-NN classifier for PSD of alpha waves and gamma waves with AUC of 0.96 and 0.93 (recall session 2). **(C)** ROC for classification for *k*-NN classifier for DWT of alpha waves and gamma waves with AUC of 1 and 1 for DWT for recall session 1. **(D)** ROC for classification for *k*-NN classifier for DWT of alpha waves and gamma waves with AUC of 0.76 and 0.76 for DWT transform for recall session 2.

The ROC is generated by plotting the TP along the *y*-axis and the FP rate along the *x*-axis. Figure 5 shows the ROC curve of the *k*-NN classifier for the PSD of alpha and gamma waves of threshold value (0.5, 1, 1) for the PSD of alpha waves, and (0.5, 0.75, 0.75) for the PSD of gamma waves, with area under the curve (AUC) values of 0.97 and 0.94 for the PSD of alpha and gamma waves, respectively, as shown in Figure 5. The ROC analysis is useful for many reasons: (1) It evaluates the discriminatory ability of continuous predictor for correctly assigning classification of two groups. (2) It gives the optimal cut-off point selection to eliminate the misclassification of two classes. (3) It shows the effectiveness of predictor. Tables 2A,B show the average of the confusion matrix for the PSD of alpha and gamma waves obtained from different iterations. From Tables 2A,B, two predicted classes for the L and NL with *n* = 31, meaning 31 subjects were tested. From the results, we observed that the classifier predicted the Ls 16 times and the NLs 15 times for the PSD of alpha waves. For the PSD of gamma waves, the predicted Ls are 15 and the NLs are 16. However, there are actually 15 Ls and 16 NLs.

**Table 2:** Confusion matrix and performance matrix of PSD of alpha and gamma band using *k*-NN classifier (recall session 2).

						(C) Performance matrix: specificity, sensitivity, and			
						accuracy for alpha and gamma waves to distinguish			
(A) Confusion matrix of alpha			(B) Confusion matrix of gamma			visual learners from non-visual learners			
*n* = 31	Predicted (NL)	Predicted (L)	*n* = 31	Predicted (NL)	Predicted (L)		Specificity (%)	Sensitivity (%)	Accuracy (%)
Actual (NL)	TN = 14	FP = 2	Actual (NL)	TN = 15	FP = 1	Alpha waves	87%	93%	96%
Actual (L)	FN = 1	TP = 14	Actual (L)	FN = 1	TP = 14	Gamma waves	93%	93%	93%

From the confusion matrix given in Tables 2A,B, the accuracy, sensitivity, and specificity are calculated. Table 2C shows the specificity, sensitivity, and accuracy corresponding to the Ls and NLs for the PSD of alpha and gamma waves.

The ROC is generated by plotting TP rate along the *y*-axis and the FP rate along the *x*-axis. Figure 5B shows the ROC curve of the *k*-NN classifier for the PSD of alpha and gamma waves of threshold value (0, 0.50, 1) for the PSD of alpha waves, and (0.5, 0.75, 0.75) for the PSD of gamma waves, with the AUC of 0.96 and 0.93 for the PSD of alpha and gamma waves, respectively. Tables 3A,B show the average of the confusion matrix for the DWT of alpha and gamma waves obtained from different iterations. From Tables 3A,B, two predicted classes for the L and NL with *n* = 34, meaning 34 subjects were tested. From the results, we observed that the classifier predicted the Ls 17 times and the NLs 17 times for the DWT of alpha waves. For the DWT of gamma waves, the predicted Ls are 17 and the NLs are 17. However, there are actually 17 Ls and 17 NLs.

**Table 3:** Confusion matrix and performance matrix of DWT of alpha and gamma band using *k*-NN classifier (recall session 1).

						(C) Performance matrix: specificity, sensitivity, and			
						accuracy for alpha and gamma waves to distinguish			
(A) Confusion matrix of alpha			(B) Confusion matrix of gamma			visual learners from non-visual learners			
*n* = 34	Predicted (NL)	Predicted (L)	*n* = 34	Predicted (NL)	Predicted (L)		Specificity (%)	Sensitivity (%)	Accuracy (%)
Actual (NL)	TN = 17	FP = 0	Actual (NL)	TN = 17	FP = 0	Alpha waves	100%	100%	100%
Actual (L)	FN = 0	TP = 17	Actual (L)	FN = 0	TP = 17	Gamma waves	100%	100%	100%

From the confusion matrix given in Tables 3A,B, the accuracy, sensitivity, and specificity are calculated. Table 3C shows the specificity, sensitivity, and accuracy corresponding to the Ls and NLs for the DWT of alpha and gamma waves.

Figure 5C shows the ROC curve of the *k*-NN classifier for the DWT of alpha and gamma waves of threshold value (0, 1, 1) for the alpha waves, and (0, 1, 1) for the gamma waves, with the AUC of 1 and 1 for the alpha and gamma waves, respectively.

Tables 4A,B show the average of the confusion matrix for the DWT alpha and gamma waves obtained from different iterations. From Tables 4A,B, two predicted classes for the L and NL with *n* = 31, meaning 31 subjects were tested. From the results, we observed that the classifier predicted the Ls 12 times and the NLs 13 times for the alpha waves.

**Table 4:** Confusion matrix and performance matrix of DWT of alpha and gamma band using *k*-NN classifier (recall session 2).

						(C) Performance matrix: specificity, sensitivity, and			
						accuracy for alpha and gamma waves to distinguish			
(A) Confusion matrix of alpha			(B) Confusion matrix of gamma			visual learners from non-visual learners			
*n* = 31	Predicted (NL)	Predicted (L)	*n* = 31	Predicted (NL)	Predicted (L)		Specificity (%)	Sensitivity (%)	Accuracy (%)
Actual (NL)	TN = 13	FP = 3	Actual (NL)	TN = 13	FP = 3	Alpha waves	81%	80%	74%
Actual (L)	FN = 3	TP = 12	Actual (L)	FN = 3	TP = 12	Gamma waves	81%	80%	74%

For the DWT gamma waves, the predicted Ls are 12 and the NLs are 13. However, there are actually 15 Ls and 16 NLs. From the confusion matrix given in Tables 4A,B, the accuracy, sensitivity, and specificity are calculated. Table 4C shows the specificity, sensitivity, and accuracy corresponding to the Ls and NLs for the DWT of alpha and gamma waves.

Figure 5D shows the ROC curve of the *k*-NN classifier for the DWT alpha and gamma waves of threshold value (0, 0.78, 1) for the alpha waves, and (0, 0.78, 1) for the gamma waves, with the AUC of 0.76 and 0.76 for the alpha and gamma waves, respectively.

Tables 5A,B show the average of the confusion matrix for the PSD of alpha and gamma waves obtained from different iterations. From Tables 5A,B, two predicted classes for the L and NL with *n* = 34, meaning 34 subjects were tested. From the results, we observed that the classifier predicted the Ls 16 times and the NLs 16 times for the alpha waves. For the gamma waves, the predicted Ls are 16 and the NLs are 16. However, there are actually 17 Ls and 17 NLs.

**Table 5:** Confusion matrix and performance matrix of PSD of alpha and gamma band using SVM classifier (recall session 1).

						(C) Performance matrix: specificity, sensitivity, and			
						accuracy for alpha and gamma waves to distinguish			
(A) Confusion matrix of alpha			(B) Confusion matrix of gamma			visual learners from non-visual learners			
*n* = 34	Predicted (NL)	Predicted (L)	*n* = 34	Predicted (NL)	Predicted (L)		Specificity (%)	Sensitivity (%)	Accuracy (%)
Actual (NL)	TN = 16	FP = 1	Actual (NL)	TN = 16	FP = 1	Alpha waves	94%	94%	97%
Actual (L)	FN = 1	TP = 16	Actual (L)	FN = 1	TP = 16	Gamma waves	94%	94%	96%

Table 5C shows the specificity, sensitivity, and accuracy corresponding to the Ls and NLs for the PSD of alpha and gamma waves.

**Figure 6:**
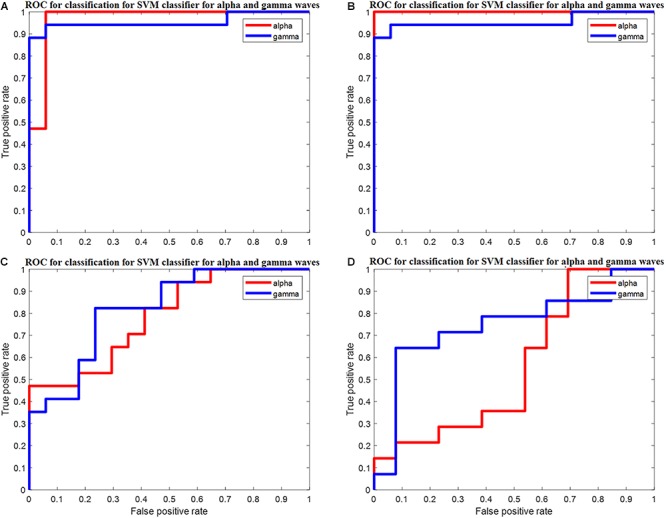
**(A)** ROC for classification for SVM classifier for PSD of alpha waves and gamma waves with AUC of 0.97 and 0.96 for PSD for recall session 1. **(B)** ROC for classification for SVM classifier for PSD of alpha and gamma waves with AUC of 1 and 0.97 for PSD for recall session 2. **(C)** ROC for classification for SVM classifier for alpha waves and gamma waves with AUC of 0.79 and 0.82 for DWT for recall session 1. **(D)** ROC for classification for SVM classifier for DWT of alpha and gamma waves with AUC of 0.56 and 0.74 for DWT for recall session 2.

Figure 6A shows the ROC curve of the SVM classifier for the PSD alpha and Gamma waves with the AUC of 0.97 and 0.96 of PSD features for recall session 1.

Tables 6A,B show the average of the confusion matrix for the PSD of alpha and gamma waves obtained from different iterations. From Tables 6A,B, two predicted classes for the L and NL with *n* = 31, meaning 31 subjects were tested. From the results, we observed that the classifier predicted the Ls 15 times and the NLs 16 times for the alpha waves. For the gamma waves, the predicted Ls are 14 and the NLs are 15. However, there are actually 15 Ls and 16 NLs.

**Table 6:** Confusion matrix and performance matrix of PSD of alpha and gamma band using SVM classifier (recall session 2).

						(C) Performance matrix: specificity, sensitivity, and			
						accuracy for alpha and gamma waves to distinguish			
(A) Confusion matrix of alpha			(B) Confusion matrix of gamma			visual learners from non-visual learners			
*n* = 31	Predicted (NL)	Predicted (L)	*n* = 31	Predicted (NL)	Predicted (L)		Specificity (%)	Sensitivity (%)	Accuracy (%)
Actual (NL)	TN = 16	FP = 0	Actual (NL)	TN = 15	FP = 1	Alpha waves	100%	100%	100%
Actual (L)	FN = 0	TP = 15	Actual (L)	FN = 1	TP = 14	Gamma waves	93%	94%	95%

From the confusion matrix given in Tables 6A,B, the accuracy, sensitivity, and specificity are calculated. Table 6C shows the specificity, sensitivity, and accuracy corresponding to the Ls and NLs for the PSD of alpha and gamma waves.

Figure 6B shows the ROC curve of the SVM classifier for the PSD of alpha and Gamma waves with the AUC of 1 and 0.97 of PSD features for recall session 2.

Tables 7A,B show the average of the confusion matrix for the DWT of alpha and gamma waves obtained from different iterations. From Tables 7A,B, two predicted classes for the L and NL with *n* = 34, meaning 34 subjects were tested. From the results, we observed that the classifier predicted the Ls 13 times and the NLs 13 times for the alpha waves. For the gamma waves, the predicted Ls are 14 and the NLs are 14. However, there are actually 17 Ls and 17 NLs.

**Table 7:** Confusion matrix and performance matrix of DWT of alpha and gamma band using SVM classifier (recall session 1).

						(C) Performance matrix: specificity, sensitivity, and			
						accuracy for alpha and gamma waves to distinguish			
(A) Confusion matrix of alpha			(B) Confusion matrix of gamma			visual learners from non-visual learners			
*n* = 34	Predicted (NL)	Predicted (L)	*n* = 34	Predicted (NL)	Predicted (L)		Specificity (%)	Sensitivity (%)	Accuracy (%)
Actual (NL)	TN = 13	FP = 4	Actual (NL)	TN = 14	FP = 3	Alpha waves	76%	76%	79%
Actual (L)	FN = 4	TP = 13	Actual (L)	FN = 3	TP = 14	Gamma waves	82%	82%	82%

From the confusion matrix given in Tables 7A,B, the accuracy, sensitivity, and specificity are calculated. Table 7C shows the specificity, sensitivity, and accuracy corresponding to the Ls and NLs for the DWT of alpha and gamma waves.

Figure 6C shows the ROC curve of the SVM classifier for the DWT of alpha and gamma waves with the AUC of 0.79 and 0.82 of DWT features for recall session 1.

Tables 8A,B show the average of the confusion matrix for the DWT of alpha and gamma waves obtained from different iterations. From Tables 8A,B, two predicted classes for the L and NL with *n* = 31, meaning 31 subjects were tested. From the results, we observed that the classifier predicted the Ls 9 times and the NLs 10 times for the alpha waves. For the gamma waves, the predicted Ls are 12 and the NLs are 11. However, there are actually 15 Ls and 16 NLs.

**Table 8:** Confusion matrix and performance matrix of DWT of alpha and gamma band using SVM classifier (recall session 2).

						(C) Performance matrix: specificity, sensitivity, and			
						accuracy for alpha and gamma waves to distinguish			
(A) Confusion matrix of alpha			(B) Confusion matrix of gamma			visual learners from non-visual learners			
*n* = 31	Predicted (NL)	Predicted (L)	*n* = 31	Predicted (NL)	Predicted (L)		Specificity (%)	Sensitivity (%)	Accuracy (%)
Actual (NL)	TN = 10	FP = 6	Actual (NL)	TN = 12	FP = 4	Alpha waves	62%	60%	56%
Actual (L)	FN = 6	TP = 9	Actual (L)	FN = 4	TP = 11	Gamma waves	75%	73%	74%

From the confusion matrix given in Tables 8A,B, the accuracy, sensitivity, and specificity are calculated. Table 8C shows the specificity, sensitivity, and accuracy corresponding to the Ls and NLs for the DWT of alpha and gamma waves.

Figure 6D shows the ROC curve of the SVM classifier for the DWT of alpha and gamma waves with the AUC of 0.56 and 0.74 of DWT features for recall session 2.

Figure 5 gives the ROC of *k*-NN classifier for PSD and DWT of alpha and gamma waves of recall session 1 and recall session 2. From Figure 5A, we observe that the PSD of alpha for recall session 1 has an AUC of 0.97, which is high. For recall session 2, the AUC slightly decreases at 0.96 but still remains high. For the PSD of gamma, the AUC is 0.94 for both sessions. This shows this model has a good class separation capacity. The model is robust since the AUC remains high for recall session 2 although it is conducted 2 months after recall session 1.

For DWT features of recall session 1 and recall session 2 the AUC is 1 and 0.76, respectively, for both DWT of alpha and gamma waves. This shows that DWT has great capabilities of class separation but does not show robustness since the AUC drops from 1 to 0.76 for recall session 2.

Similarly, Figure 6 shows the SVM classifier for PSD and DWT of alpha and gamma waves of recall session 1 and recall session 2. Here the PSD of alpha and gamma has the AUC of 0.97 and 0.96 for recall session 1 and AUC of 1 and 0.97 for recall session 2. This shows about more the robustness of PSD is class separation since the AUC values remain high for both sessions, although the classifier has been changed.

For DWT of alpha and gamma the value of AUC is 0.79 and 0.82 for recall session 1 and 0.56 and 0.74 for recall session 2. Here, also the AUC is slightly high in case of recall session 1. Compared to PSD, the AUC values for DWT are much lower and also the decrease between recall session 1 and recall session 2 is more considerable. That show that DWT has less class separation capabilities and is less robust.

The results show that PSD feature can be more reliably used for distinguishing visual learners from visual non-learner.

## Discussion

**Figure 7:**
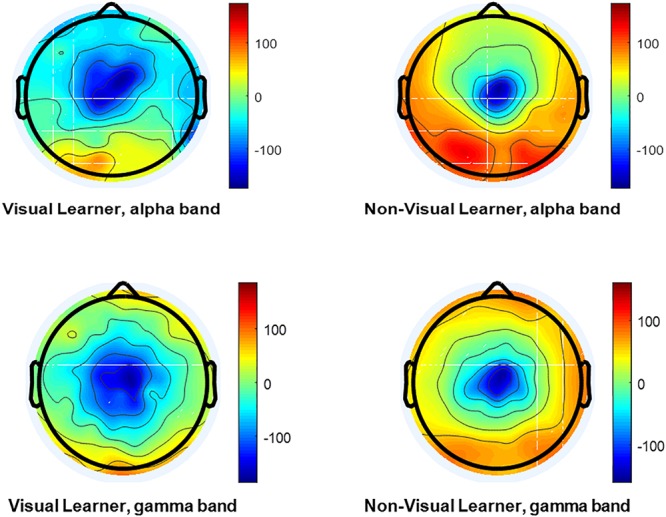
The topographical map of a visual learner and non-visual learner. Brain wave activation was found very high in left posterior temporal and left frontal region in visual non-learner. However comparable activation, but lower in visual learner scenario.

In this study, the learning styles are studied by analyzing the EEG signals, and the machine-learning classifier is used for the classification of visual learners from non-visual learners (Figure 7).

According to the authors’ knowledge, none have distinguished a visual learner from a non-visual learner using video contents as stimuli along with EEG signals. Human has exceptional learning abilities, which allows individuals at learning stage to adapt to different learning environment. However, there is a difference in learning abilities of individual which become obvious when they progress in life. There are different models and learning theories that explain how individual learns (Stern, 2017). Many researchers have reported findings based on learning theories to identify the learning style of the learners. The Felder and Silverman theory is one of the learning theories that is used by most studies that explore the learning style of the learners (Klašnja-Milićević et al., 2017). In Marschark et al. (2013) and Kim et al. (2015), the researchers explored static media, such as pictures and words, to categorize the visual learners and the verbal learners. In these studies, they accurately classified a visual learner from a verbal learner based on the decreased value of theta, and the increased value of beta in the case of a visual learner, and vice versa for a verbal learner. Their drawback is that the analysis is based on static media, which does not include all the aspects of visual modality. However, in our study, videos content (dynamic media) is used to include all the aspects of visual modality along with studying the brain responses via EEG recordings and thus eliminating the drawback of the above-mentioned studies (Kim et al., 2015).

Recent studies based on Kolb’s model, another learning theory based on the learner’s internal cognitive processes, used an online test for the subjective measure, and EEG with only the eyes closed condition (Ali et al., 2016). Kolb’s model with the EEG eyes closed condition does not fully represent the relationship with a visual learner because the online questioner asks the students to select their suitable learning style according to their viewpoint, which is the main limitation of Kolb’s theory (Kelly, 1997). Kolb’s test is based solely on the way learners rate themselves. It does not rate the learning style preferences through standards or behaviors. The reported findings were based on the resting state of the EEG recordings, which does not explain the whole picture of the neuronal responses during learning and/or during the retrieval of learned information. The present study recorded the EEG during the resting states, learning tasks, and information retrieval tasks. Thus, in this study, the decision of being a visual learner or non-learner is based on the neuronal responses recorded during the information retrieval tasks combined with the machine-learning algorithm, rather than using a subjective questionnaire, such as one reported in the resting state EEG (Ali et al., 2016).

As mentioned above, the existing studies do not used videos as a stimulus to classify the visual learning style, especially in engineering disciplines. Identifying a learner as a visual learner using videos is an important component of the visual modality. Previous results are based on only one component of the Felder theory, which is visual-verbal, as reported in Felder and Silverman (1988). All these previous works neglect the important aspect of the visual learning modality, such as videos (dynamic media), and focus only on pictures and words (static media).

Considering these limitations, we have attempted to present a classification-based model to identify visual learners using video (dynamic media) contents for learning; videos are more reliable and include all the aspects of visual learning modalities and is a more realistic way of presenting information in a relatable manner. Here, the EEG recordings are recorded while performing the learning and memory tasks. We have computed the PSD and DWT for the alpha and gamma frequencies of the EEG, recorded during the learning tasks and retrieval tasks and discriminated the subjects between groups, i.e., visual learners and non-learners. Since, we are distinguishing visual learner and non-visual learner. A separate analysis of alpha and gamma waves is required. That’s why we did not use all the frequency band together. But we saw the effect of alpha-gamma together. The results show that combining alpha-gamma did not increase the accuracy of our classification model. According to Arnaldo et al. (2018), variations are observed in the alpha and gamma bands during cognitive tasks, such as the visualization of learning tasks. The results of our study identified a methodology to classify visual learners from non-visual learners. This study confirmed that the learning styles of individuals influence the neuronal electrical potentials generated during the learning and retrieval of learned information. Based on our results, we conclude that subjects who are visual learners learned better using videos (dynamic media), which is easier to understand and relatable. Thus, the further development of learning modalities can be considered as future work. In addition, for further studies, the authors believe that the brain neuronal signals will facilitate the understanding of the impact of changes in the learning modalities.

### Limitations of the Study

The study has few limitations. The small sample size is not enough to predict the learning style of the students. However, future studies can be conducted to predict learning style of the students. In addition, this study investigates the learning style of university students only. Finally, the learning material used in this study was related to human anatomy and physiology contents; thus, learning style cannot be generalized to link with learning ability of all types of academic learning contents or memory recall ability. Also, EEG is the only modality we use to record the brain signals. Although EEG is considered one of the favorable methods it has few caveats. It has excellent temporal resolution but poor spatial resolution. Volume conduction is also one of the limitations.

## Conclusion

In this work, we have proposed a brain-learning model for identifying visual learners and non-visual learners using EEG signals. The neural activity of the subjects is observed in all regions of the brain. Three different features are extracted and tested to find the stabilized features for the task in hand. To distinguish the visual learners from the non-visual learners, the PSD and DWT features are extracted over 16 scalp sites such as frontal, occipital, and parietal regions which play active role during visual learning, and fewer features were selected using PCA to train and test the classifier. The *k*-NN classifier with the Mahalanobis distance and SVM are used to classify the visual learners and non-visual learners. The classification accuracies were recorded as 97% and 94% for the PSD alpha and gamma bands for the recall session 1, and 96% and 93% for the recall session 2, for the visual learners and non-visual learners, respectively. For DWT features using *k*-NN classifier 68% and 100% accuracy rate for recall session 1 and 100% accuracies rate for the recall session 2 for the alpha and gamma band is recorded. For PSD alpha and gamma band 97% and 96% accuracies rate for the recall session 1, 100% and 95% accuracies rate for recall session 2 using SVM classifier are reported. Similarly 79% and 82% accuracy for recall session 1 and 56% and 74% accuracy for recall session 2 for DWT features using SVM classifier are reported. From above results, we concluded that PSD for SVM shows the best results for this study.

The EEG alpha and gamma bands showed good agreement with the learning process. The analyses of these frequency bands indicate the clear difference between the visual learners and non-visual learners. Therefore, our proposed brain-learning model will be helpful in academic applications, such as to help university students to identify visual learners and non-visual learners. This work can be extended by conducting further experiments to explore other learning modalities, such as audio and kinesthetic, using EEG signals.

## Ethics Statement

The protocol of this study was approved by the ethics coordination committee, Universiti Teknologi PETRONAS, Malaysia, and by the Human Research Ethics Committee of the Universiti Sains Malaysia.

## Author Contributions

HA and AM designed the study protocol and collected the data. SJ and IF pre-processed and analyzed the EEG signals. All authors contributed in the preparation of the manuscript and reviewed it.

## Conflict of Interest Statement

The authors declare that the research was conducted in the absence of any commercial or financial relationships that could be construed as a potential conflict of interest.

## Footnotes

1 https://www.reference.com/education/advantages-disadvantages-visual-learning-5ede107612d4e80

2 http://www.vfirst.com/blog/techfirst/dimension-reduction-techniques-pca-vs-lda-in-machine-learning-part-2/
